# Recombinant vector vaccine evolution

**DOI:** 10.1371/journal.pcbi.1006857

**Published:** 2019-07-19

**Authors:** James J. Bull, Scott L. Nuismer, Rustom Antia

**Affiliations:** 1 Department Integrative Biology, University of Texas, Austin, Texas, United States of America; 2 Department of Biological Sciences, University of Idaho, Moscow, Idaho, United States of America; 3 Department of Biology, Emory University, Altanta, Georgia, United States of America; Eötvös Loránd University, HUNGARY

## Abstract

Replicating recombinant vector vaccines consist of a fully competent viral vector backbone engineered to express an antigen from a foreign transgene. From the perspective of viral replication, the transgene is not only dispensable but may even be detrimental. Thus vaccine revertants that delete or inactivate the transgene may evolve to dominate the vaccine virus population both during the process of manufacture of the vaccine as well as during the course of host infection. A particular concern is that this vaccine evolution could reduce its antigenicity—the immunity elicited to the transgene. We use mathematical and computational models to study vaccine evolution and immunity. These models include evolution arising during the process of manufacture, the dynamics of vaccine and revertant growth, plus innate and adaptive immunity elicited during the course of infection. Although the selective basis of vaccine evolution is easy to comprehend, the immunological consequences are not. One complication is that the opportunity for vaccine evolution is limited by the short period of within-host growth before the viral population is cleared. Even less obvious, revertant growth may only weakly interfere with vaccine growth in the host and thus have a limited effect on immunity to vaccine. Overall, we find that within-host vaccine evolution can sometimes compromise vaccine immunity, but only when the extent of evolution during vaccine manufacture is severe, and this evolution can be easily avoided or mitigated.

## Author summary

Recombinant vector vaccines are live replicating viruses that are engineered to carry extra genes derived from a pathogen—and these extra genes produce proteins against which we want to generate immunity. These vaccine genomes may evolve to lose the extra genes during the process of manufacture of the vaccine or during replication within an individual, and there is a concern that this evolution might severely limit the vaccine’s efficacy. The dynamics of this process are studied here with mathematical models. The potential for vaccine evolution within the host is somewhat limited by the short-term growth of the vaccine population before it is suppressed by the immune response. We find that evolution is a problem only when the process of manufacture results in the majority of the vaccine virus being revertant. We show that increasing the vaccine inoculum size or reducing the level of revertant in the vaccine inoculum can largely avoid the loss of immunity arising from evolution.

## Introduction

Live vaccines replicate within the host. As true of any reproducing population, these within-host vaccine populations may evolve. For live vaccines that do not transmit, any within-host evolution is a dead end and might thus seem to be irrelevant to vaccine function. But if the process is fast enough, or the vaccine population replicates long enough, the vaccine population may evolve to a state where it is ineffective or virulent—either change would be bad.

The two main types of live viral vaccines are attenuated and recombinant-vectored. Most live virus vaccines in use today are attenuated, their reduced virulence typically achieved by adapting the wild-type virus to a new environment (e.g. replication in a novel cell line or low temperature), with a consequent reduced replication rate in humans. The use of attenuated vaccines is too risky for pathogens such as HIV, and a safer alternative is to develop a live, recombinant vector vaccine where one or a few pathogen genes with immunogenic activity (proteins that elicit protective immunity) are expressed from a benign virus vector.

The expected consequences of within-host evolution differ between these two types of vaccines (Table 1). Evolution of an attenuated vaccine is likely to be a reversion toward the wild-type state, the rate of this process depending heavily on vaccine design and the duration of vaccine virus replication in the host (reviewed in [1]). To a first approximation, reversion toward the wild-type state should lead to the vaccination more closely resembling natural infection [2], such as higher virus densities, side-effects and disease, and possibly an increased immune response. Within-host evolution of an attenuated vaccine might also predispose the virus to better transmission—also reflecting the wild-type state—but this outcome is not assured: viral adaptation to different tissues within the host may hamper growth in and dissemination from tissues important in transmission (e.g., [3]).

**Table 1:** Consequences of evolution for traditional live attenuated and recombinant vector vaccines.

Factor	Attenuated vaccine	Recombinant-vector vaccine
type of evolution	reversion toward wild-type	loss of insert
virulence	higher virulence	little change in virulence
immunity	possible increase	possible reduction
transmission	increase	no effect or increase

The expected consequences for evolution of a recombinant-vectored vaccine are fundamentally different [4]. In most cases, the antigen against which immunity is sought comes from a foreign transgene inserted into a competent viral vector without replacing any vector genes. Vectors in development include adenovirus, VSV (vesicular stomatitis virus) and CMV (cytomegalovirus). The vector genome carries out all viral amplification and transmission functions, and the transgene does not contribute to any process benefiting vector reproduction. From an evolutionary perspective, the transgene is both dispensable and potentially costly: selection may favor loss of the transgene and thus loss of vaccine’s ability to elicit immunity against the antigen encoded by the transgene. This evolution therefore generates something akin to infection by the wild-type vector. As vectors are typically chosen to be avirulent for immune competent hosts, vaccine evolution will result in no more than a harmless infection that does not generate immunity to the antigen encoded by the transgene.

Considerable attention has recently been given to the evolution of attenuated vaccines and designs that retard their evolution. Evolutionary stability of attenuated vaccines seems attainable by engineering designs, including the introduction of hundreds of silent codon changes, genome rearrangements, and some types of deletions (comparisons and reviews are provided by [1, 5, 6]). Far less thought has gone into the consequences of evolution for recombinant vector vaccines or of strategies to minimize this evolution.

Although recombinant vector vaccines are not yet in widespread use, many are under development [7, 8], and their success may rest on understanding within-host evolution. Here we explore how the combination of evolution during the process of vaccine manufacture and during its within-host dynamics following vaccination could affect the immune responses elicited by a recombinant vector vaccine and reduce its efficacy—the specific interaction between evolution and immunity. We consider viral vaccines and focus on vaccines that cause short-duration (acute) infections. The ideas we discuss also apply to live vaccines of bacteria and other pathogens.

Our overall message is that while vaccine evolution may occur, it is either unlikely to be a problem (i.e., compromise the generation of immunity), or it is easily mitigated. When vaccine evolution does limit the adaptive immune response, we identify ways of escaping such outcomes. Our analysis rests on mathematical models, but most results can be explained intuitively (perhaps only in hindsight), with the main results illustrated graphically; many analyses are relegated to Supporting Information. Our analysis assumes that vaccines replicate within the host untill cleared by host immunity; we exclude vaccines that reproduce for just a single infection cycle (e.g., Modified Vaccinia Virus Ankara), as they have no significant opportunity for evolution.

## Methods

Our models are numerical analyses of ordinary differential equations. The equations are given in supporting information (S1 Appendix). The were numerically evaluated and graphed in R (S1 File, a Markdown file), sometimes also evaluated in Mathematica (S2 File).

## Results

### Why the problem is not simple

The key question is whether evolution of the vaccine virus (henceforth just ‘vaccine’) meaningfully affects immunity to the antigen encoded by the foreign transgene (henceforth just ‘antigen’). The potential for vaccine evolution is easy to understand. Through mutation, any large vaccine population will contain mutants that inactivate or delete the foreign transgene, and those revertants will then grow amidst the vaccine. Vaccine inferiority may accrue in two different ways: the transgenic insert and its expression may intrinsically impair vaccine growth, and adaptive immunity to the foreign antigen may impair the vaccine’s growth but not the revertant’s during an infection.

It is easy to appreciate how and why the vaccine may be inferior to the revertant, and this can result in an increase in frequency of the revertant. However, the relationship between this evolution and the extent of immunity to the vaccine antigen is more complex. We thus explain some of the factors that affect how this evolution translates into a reduction in immunity to the antigen, and why in some circumstances, substantial evolution can result in little change in immunity to the antigen, while in different situations it can result in a substantial reduction.

#### Two realms of vaccine evolution

**Fig 1:**
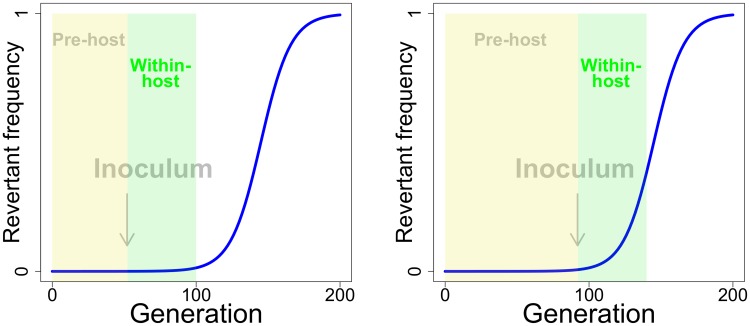
Impact of pre-host evolution on within-host evolution. The blue curve depicts the time course of revertant frequency in competition with vaccine, where the revertant has a 10% fitness advantage and starts at a frequency of 10^−6^. The curve shows the well-known population genetic principle that, while the favored type (revertant) is rare, its absolute frequency changes very little. But the frequency eventually reaches a level at which evolution is rapid. The yellow box represents a possible period of pre-host evolution, the green box representing the period of within-host evolution. The periods of within-host evolution are drawn to be the same length in right and left panels, as if the vaccine has the same within-host duration in both cases. The arrow represents a possible point at which manufacture would end and an inoculum be created, thus defining the boundary between pre-host and within-host evolution. The left panel depicts a short period of vaccine manufacture, the right a longer period of vaccine manufacture and one in which more pre-host evolution has occurred. It is thus easy to see the potential importance of pre-host vaccine evolution on within-host evolution, for even when the revertant is not a large component of the inoculum, it can be poised for rapid evolution within the host (right panel). The curve obeys $p_{t}=\frac{p_{0}w^{t}}{p_{0}w^{t}+1-p_{0}}$, in which *p*_*t*_ represents the revertant frequency in generation *t* and *w* the fitness of revertant relative to vaccine. The curve is drawn for a common evolutionary process across pre-host and within-host evolution, but evolution in the within-host phase will typically experience different parameters than evolution in the pre-host phase.

Vaccine evolution can be inimical to immunization by limiting vaccine antigen levels in the host. As noted above, one realm in which evolution may occur is within the host, starting with the inoculum and ensuing until vaccine clearance. A second realm of evolution affecting antigen levels is that occuring during manufacturing—during growth of the virus to prepare the stock used for inoculation. Both realms are part of a continuum, any evolution during manufacturing advancing evolution within the host (Fig 1). The inoculum sets the starting point for within-host evolution, and indeed, an inoculum that is mostly revertant will limit vaccine efficacy even if no further evolution occurs within the host. From population genetics principles [9], even if the inoculum has a seemingly low frequency of revertant, the evolution that has occurred prior to inoculation may greatly accelerate within-host evolution (Fig 1). Paradoxically, when considerable evolution occurs in manufacture, such that the inoculum is primarily revertant, there is little opportunity for further evolution within the host—the damage is already done.

The effect of any ‘pre-host’ evolution on the host immune response is potentially as important as the effect of within-host evolution. An important difference between the two realms is that pre-host evolution may be more easily mitigated than is within-host evolution. That is, controlling pre-host evolution may be a feasible way to limit within-host evolution and to limit the loss of immunity from vaccine evolution.

Since pre-host and within-host evolution represent different realms on a continuum, we adopt a language that attempts to distinguish them and avoid confusion. We use the following:

- ‘Pre-host evolution’ refers to evolution during manufacture that affects inoculum composition
- ‘Within-host evolution’ refers to evolution that occurs within the host after inoculation
- ‘Evolution’ refers to either or both of the above.

It is easily appreciated that the specifics of evolution in the two realms may differ—vaccine growth and fitness in an *in vitro* environment (pre-host) will often differ from that within the host. Regardless, however, any pre-host evolution will advance subsequent within-host evolution, unless the revertant is selected in opposite directions pre-host and within-host. There may even be a common molecular basis to vaccine inferiority in both the pre-host and within-host environments that will render the two processes somewhat similar (see below).

#### A short duration of infection limits within-host evolution

**Fig 2:**
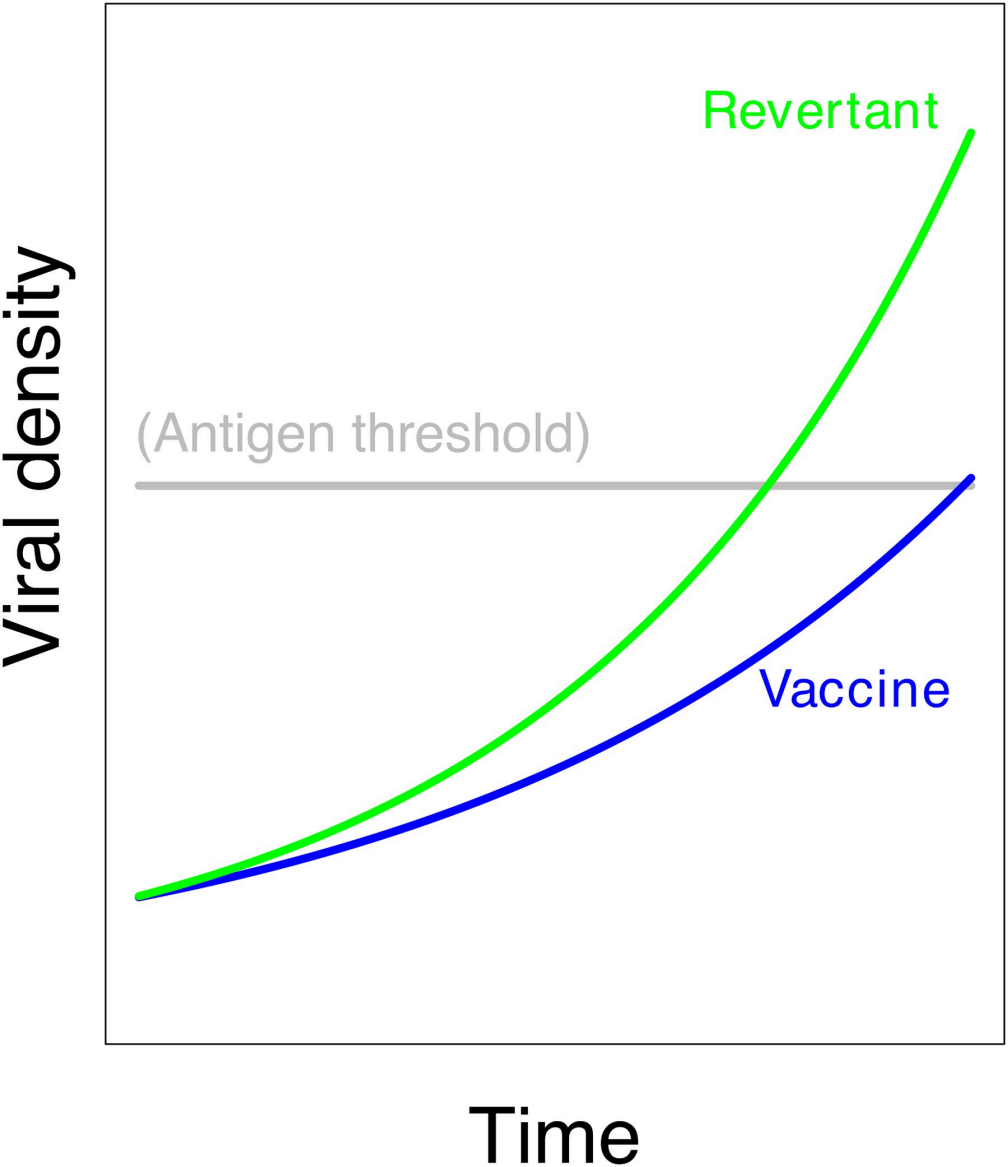
Independent growth of vaccine (blue) and revertant (green). The revertant virus has the superior growth rate, but in the absence of interference between the two, vaccine growth is unimpeded and immunity is triggered.

Any fitness advantage of revertant means that its frequency—its abundance relative to the vaccine—will increase during active viral growth (Fig 2). However, when the infection caused by the vaccine is acute, as we consider here, the magnitude of possible within-host evolution is limited by the short duration of viral growth before clearance. If the inoculum is largely free of revertant, even a moderate fitness cost of the vaccine may have little effect on vaccine evolution, such that vaccine fitness effects and evolution can be ignored.

#### Evolution versus immunity

Surprisingly, vaccine evolution *per se* need not reduce the immune response, even when its magnitude is large. If overgrowth by revertant does not interfere with vaccine growth, then vaccine growth and antigen production are not affected (Fig 2). Evolution affects antigen production only to the extent that revertant superiority suppresses vaccine growth and thereby suppresses antigen production.

#### Numbers versus frequencies

Models of evolution often address relative frequencies, on a scale of 0 to 1. Immunity develops in response to vaccine density. In addressing immunity, it is thus necessary for the model to track densities, whereas any evolution is more easily described with frequencies (the two approaches can be compared between Figs 1 and 2).

The challenges are thus to understand (i) when and how much vaccine evolution occurs; (ii) whether and to what extent evolution affects the abundance of vaccine virus in the host over time; and (iii) how changes in vaccine abundance affect the generation of adaptive immunity against the antigen. The arguments presented above are qualitative and only superficially identify the scope of the problem. Quantitative understanding ultimately rests on analysis of mathematical models. However, as the models have many interacting processes—minimally innate immunity, adaptive immunity and intrinsic growth differences between vaccine versus revertant—we first verbally explain the biology underlying the processes that go into those models.

### Bases and consequences of vaccine inferiority and interference

#### Intrinsic fitness differences

Intrinsic fitness effects are considered here to be those that stem from the intracellular processes of viral gene expression and assembly, independent of host immune responses. Intrinsic fitness differences between the vaccine and the revertant (wild-type vector) are plausible because the transgene is non-essential and has no evolutionary history with the vector genome. Thus, the insertion may be disruptive, and the resulting antigen expression may interfere with vector functions. Intrinsic fitness effects are expected to affect evolution during vaccine manufacture as well as within-host evolution, but it has largely been investigated *in vitro*, as would apply to manufacture and the pre-host phase. Indeed, intrinsic fitness differences may be the sole or at least the most important bases of vaccine inferiority. Because recombinant viruses have often been observed to evolve loss or down-regulation of engineered inserts, they are now commonly observed during *in vitro* growth for their ‘genetic stability’ (e.g., [10–22]). Some recombinant viral genomes are stable over short term transfers in culture, others not, indicating that intrinsic fitness effects of the engineering are not universal. Thus, the possibility of vaccine inferiority should not be ignored, and furthermore, even when a vaccine appears to be stable over a few transfers, the short term population retention of antigen expression may mask an underlying long term instability. Thus most observations of stability merely set limits on the possible magnitudes of inferiority. Yet even if vaccine selective ‘neutrality’ turns out to be fleeting, merely a mistaken impression from short-term observations, we will find that the phenomenon of short-term stability mirrors a solution to minimize vaccine evolution within the host—the solution of limiting vaccine growth.

#### Three mechanisms of vaccine-revertant interference

Fig 2 presented a hypothetical case in which evolutionary superiority of revertant did not suppress vaccine growth, hence evolution had little effect on antigen production. That process was one in which there was no interference between vaccine and revertant growth. Evolution does become important to antigen levels if vaccine and revertant interfere so that vaccine growth is depressed by the revertant, or if the duration of infection by the vaccine strain is reduced. In either case the revertant will then suppress antigen levels. Again, the problem is complicated by the limited duration of the infection: reduced antigen production due to vaccine evolution depends not only on interference between the two genomes but also on overall growth and the extent to which it affects the level of immunity to vaccine and vector. A mechanism that forces interference between vaccine and revertant can also limit the total amount of viral growth, thereby limiting evolution.

Evolution of vaccine versus revertant thus depends on details, in particular, the specific mechanism by which revertant interferes with vaccine growth. We describe three different mechanisms that have been proposed that may be relevant to vaccine competing with revertant: innate immunity, resource limitation, and adaptive immunity to the vector backbone shared by the vaccine and revertant virus. (These are not the only possible mechanisms of within-host parasite competition [23], but they capture the relevant immune processes.) For many vaccines, each mechanism will impede revertant and vaccine equally as a collective population, thus ensuring interference.

It was initially believed, implicitly if not explicitly, that the adaptive immune response played the dominant role in the control of viruses and other infections. In the 1990’s, Janeway and Medzhitov identified shared pathways for the control of pathogens between vertebrates and *Drosophila*, even though *Drosophila* lacks an adaptive immune response (reviewed in [24]). This led to a resurgence of interest in the role of innate immunity in the initial control of infections. Later modeling studies of influenza infections suggested yet another mechanism, that the initial control of these infections could be largely described by simple resource limitation models, of the type used in ecology for population growth [25, 26]. The realization that all three different processes might suppress viral infection led to more careful examination of the roles of different factors in the early control of acute infections [27–30]. The relative role of each mechanism in clearing infections is the basis of ongoing discussion, but it is widely accepted that the roles differ among infections by different viruses and that each mechanism is potentially important for some viruses.

- **Innate immunity**. There are two broad arms of immunity for suppressing vaccine growth within the host, the innate and the adaptive immune responses. Innate immunity is triggered by conserved molecules associated with pathogens (Pathogen Associated Molecular Patterns, [24]). Conserved structures of pathogens targeted by innate immunity include dsRNA, frequently accompanying viral replication, plus lipopolysaccharides and endotoxins of bacteria [31]. Because innate immunity involves the activation of a standing population of immune cells such as macrophages and dendritic cells, or triggering of the complement pathway, it can be elicited much more rapidly than the adaptive response; the latter requires many rounds of clonal expansion of rare antigen-specific cells to generate a population large enough to control the infection [32]. Furthermore, recent studies have shown that the innate response is required for the initial stimulation of the adaptive response [33]. Thus, innate immunity has a major role in early control of the viral population. Innate immunity can control both vector and vaccine, and it is not likely to discriminate between two genomes that differ by a single, non-essential gene (the transgene).
- **Resource limitation** Another mechanism by which revertant levels can suppress vaccine levels is resource limitation. Both the vaccine and revertant virus use the same resource (susceptible host cells). Resource limitation can control the infection if the virus depletes this resource, whereby the rate of virus output falls below its intrinsic death rate [25]. Like innate immunity, resource limitation is expected to affect vaccine and revertant similarly. Resource limitation has been considered an important mechanism for competing malarial strains within the host [34, 35].
- **Adaptive immunity** Adaptive immunity can be induced by the revertant and the vaccine virus. Adaptive immune responses to antigens expressed by the revertant will presumably affect the vaccine and revertant equally—because the vaccine encodes a complete vector genome, and the revertant is also a complete vector/virus. As with the preceding pair of mechanisms, adaptive immunity elicited by the revertant will also depress the abundance of the vaccine virus. Adaptive immunity to the vaccine antigen will be considered shortly.

All three interference mechanisms will potentially operate in any vaccinated host. With all three operating, one mechanism may take precedence over the others, simply because it is activated earlier or enforces a lower limit on viral density than the others. However, there are different stages or degrees of vaccine suppression, so an early mechanism may act to control the infection without clearing it, and another mechanism may act later to clear. Because of the delay in developing an adaptive response, viral suppression by adaptive immunity typically occurs later than effects of innate immunity or resource limitation and so might seem to be unimportant in vaccine evolution. Yet adaptive immunity may be important in clearing the vaccine following control by other mechanisms, in which case it could have an important role in vaccine evolution.

#### Adaptive immunity to the vaccine antigen may also contribute to vaccine inferiority–and feed back to inhibit itself

The preceding paragraphs omitted adaptive immunity to the antigen. By its very nature, adaptive immunity suppresses vaccine growth. But adaptive immunity to the antigen is specific to the vaccine and is thus another reason—besides intrinsic fitness effects—that the vaccine may have lower fitness than revertant. The evolutionary consequences should be the same for both types of inferiority, reducing the long term generation of antigen levels within the host, but adaptive immunity would be irrelevant to vaccine evolution during manufacturing and during early growth within the host. An interesting twist is that adaptive immunity to the antigen might eventually feed back negatively on itself to limit its own growth—immunity against a virus is intrinsically inhibitory, so adaptive immunity against the vaccine will limit vaccine growth and thus limit antigen build-up that would fuel further immunity. One question is whether this self-inhibition is worsened with vaccine evolution.

The effect is biologically complicated because adaptive immunity to the antigen does not necessarily translate into selection against the vaccine. Selection against the vaccine *per se* operates only when adaptive immunity specifically targets the vaccine genome over the revertant genome, and this selection need not occur—either because adaptive immunity is so delayed that it is never manifest during vaccine growth, or because the antigen is physically decoupled from its genome when attacked by the adaptive response. Without imposing selection on the vaccine, antigen-directed immunity will not affect vaccine evolution.

### Beyond intuition: A formal model and numerical results

**Fig 3:**
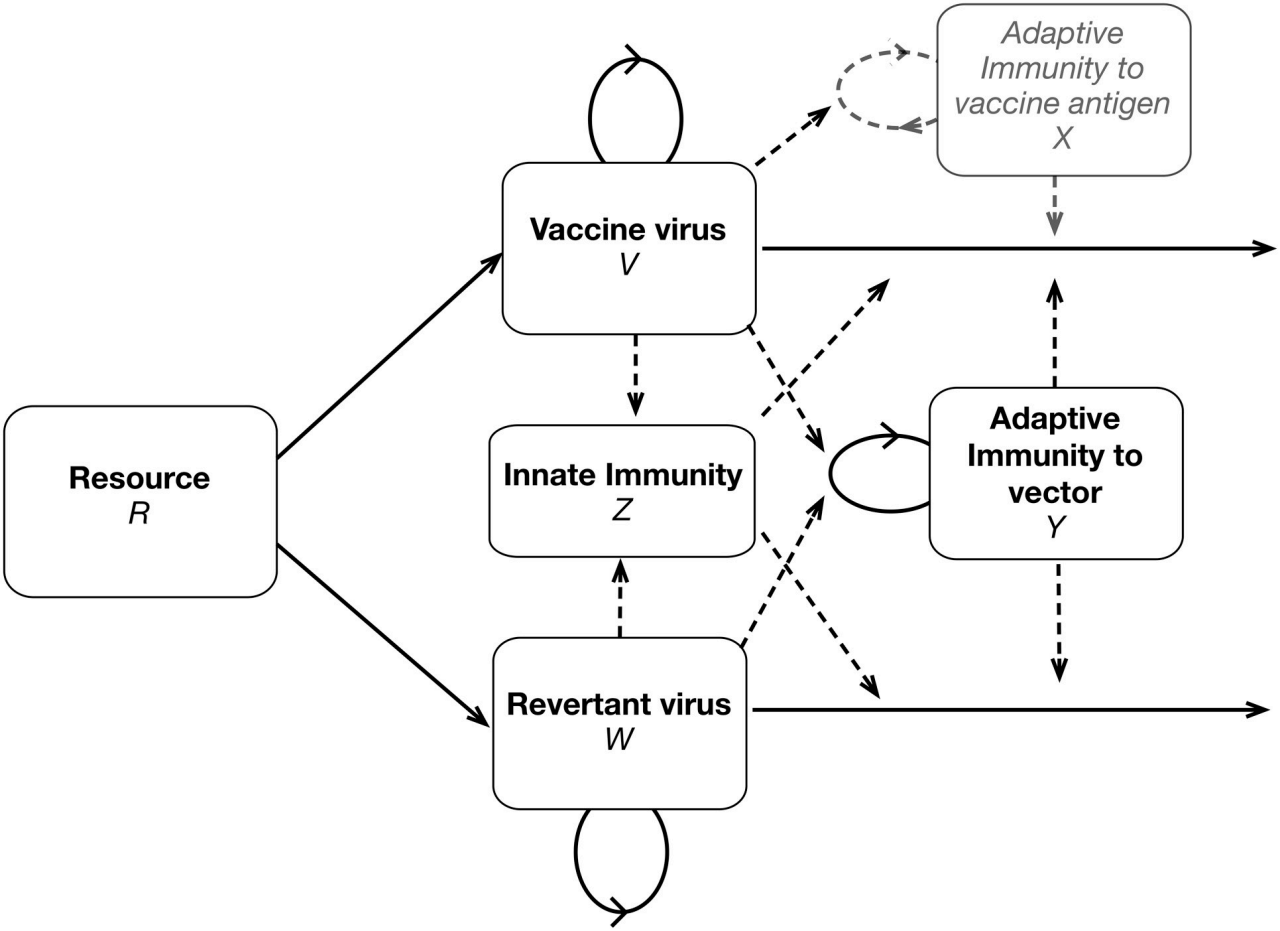
Diagram of model processes and interactions. This figure gives all the processes in the full model that includes resource limitation with innate and adaptive immunity. Solid lines represent variables (V, W, R, Z, X, and Y) and dashed lines represent influences. Note that only the top-most box in gray, the specific immune response to the vaccine antigen, acts differentially on the vaccine vs revertant virus. Not all of these components are included in each iteration of the model. Furthermore, this figure omits pre-host processes that occur during vaccine manufacturing that affect inoculum composition.

We now employ quantitative models to evaluate the intuitive ideas presented above. Given the high dimensionality of the problem, we are especially interested in how well intuition works and whether generalities are observed across large regions of parameter space. A flow diagram of the elements and interactions within the host reveals the complexity of the model (Fig 3) and facilitates understanding the dynamical equations. *V* and *W* are the respective vaccine and revertant densities, with intrinsic growth and death rates governed by four parameters (not illustrated). The model also includes variables for resources (*R*), innate immunity (*Z*), adaptive immunity to vector (*Y*), and adaptive immunity to antigen (*X*) that are both influenced by and influence *V* and *W*. In the following sections, we explore the dynamics of these interactions with simulations and present results graphically (the results presented do not allow resource limitation to influence dynamics; trials where resource limitation matters were conducted but are not shown). Equations and parameter values are provided in S1 Appendix. Resource limitation and innate immunity yield qualitatively similar results, so trials with resource limitation are not illustrated in the main text. The equations apply only to within-host processes; any pre-host evolution is subsumed into inoculum composition.

The models assist us by forcing us to specify assumptions for how the viruses and immunity interact, and by allowing us to rigorously explore outcomes in different scenarios. However, there is uncertainty in the model structure, many parameter values are unknown, and different viruses will behave somewhat differently. Consequently, we focus on broad generalities that arise from many simulations and illustrate these for a few specific cases, reserving Supporting Information files for further details. The presentation below briefly discusses the dynamics of individual trials for illustration but then moves to contour plots that reveal differences in outcomes as the key parameters are changed. The model used here incorporates the structure of earlier models that described immune responses [36–38]; parameter values used here were chosen as described in some of these earlier studies.

#### Evolution can matter

**Fig 4:**
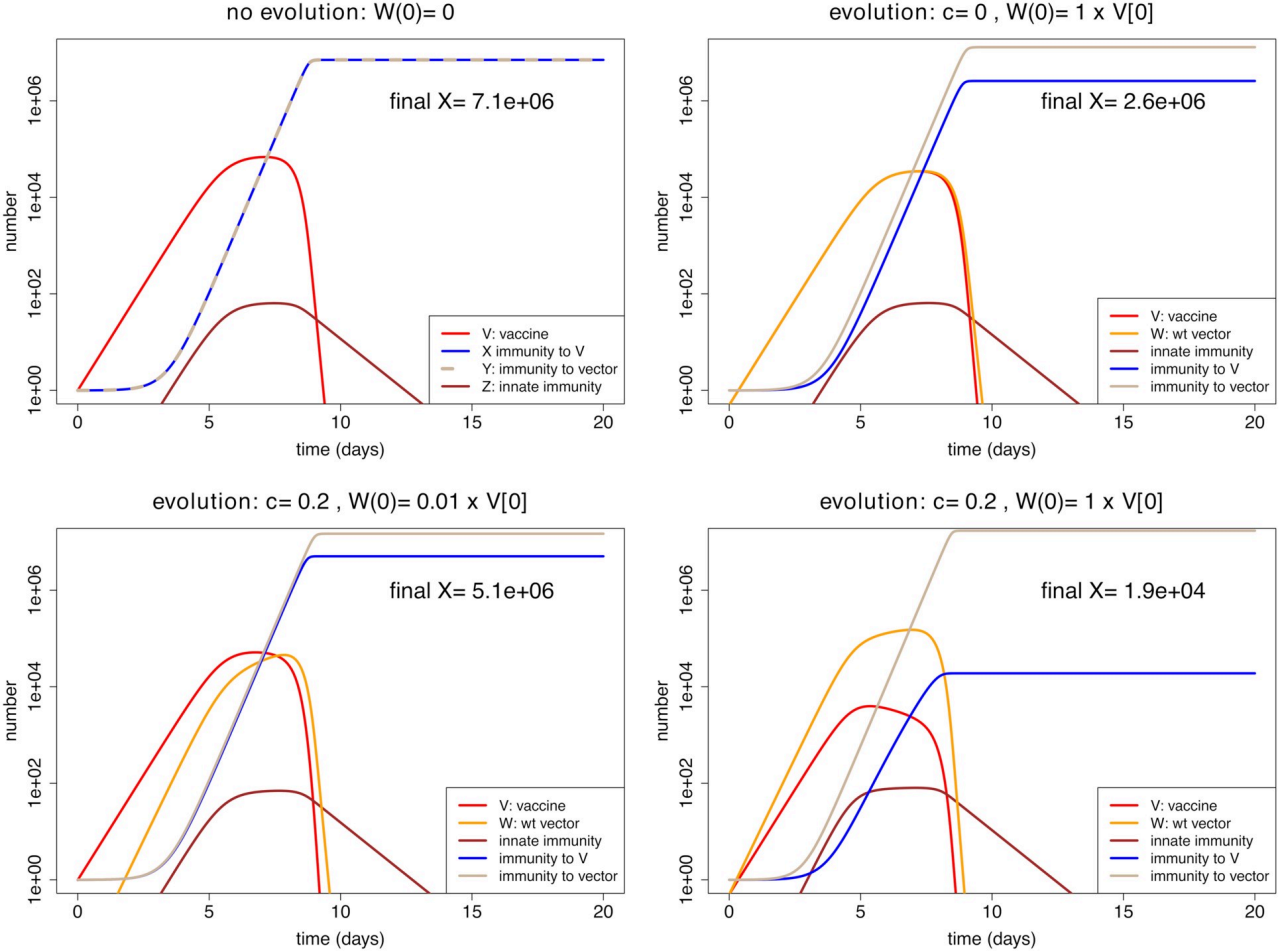
Representative dynamics contrasting vaccine evolution with no evolution. The combination of intrinsic fitness effects and revertant abundance in the inoculum has the most profound effect in depressing immunity, but depressions are observed even when any evolution is allowed. The vertical axes use a log scale, thus diminish the visual appearance of changes. (Top left): Absence of revertant (i.e. no pre-host or within-host evolution). (Top right): The revertant is included at half the inoculum (representing pre-host evolution); it has no intrinsic fitness advantage over vaccine. Immunity (to vaccine) is reduced to just over a third of the level with no evolution. (Lower left): The revertant is a small fraction of the inoculum (0.01) but it has a 20% fitness advantage over vaccine. The level of immunity is 71% that with no evolution. (Lower right): The revertant is half the inoculum and has a 20% fitness advantage over vaccine. The level of immunity is now less than 0.3% that with no evolution—a depression of almost 3 orders of magnitude. The trials are parameterized so that virus is controlled by innate immunity with final clearance due to adaptive immunity; the mutation rate is 0 in all cases. Equations, initial conditions and parameter values not shown here are given in S1 Appendix; R code is included in S1 File.

In the trials used for illustration, we allow innate immunity to control the infection and adaptive immunity to cause final clearance. Such a scenario might correspond to the dynamics of *Listeria* or influenza infections of mice [32], or the early dynamics of SIV infections [39]. To get a sense of the full dynamics in the model, we show the time course of dynamics for the different variables (Fig 4) under conditions of no evolution (top left), just pre-host evolution—revertant abundant in the inoculum but with no fitness advantage (top right), mostly within-host evolution (bottom left), and both (bottom right). The effect of different types of evolution is seen from a comparison of the panels. Our chief interest is in final immunity to vaccine, the blue curves.

The most visible effect of evolution on immunity to the antigen is evident in the lower right panel, which combines pre-host evolution with a fitness advantage of the revertant. However, the log scale diminishes the visual impact of substantial evolution in other cases. When the revertant is half the inoculum but has no fitness advantage, the immune response is diminished by nearly 3-fold (top right). Overall, the impression is that one must at least suppress either pre-host or within-host evolution to avoid a large loss in immunity (lower right versus the others).

**Fig 5:**
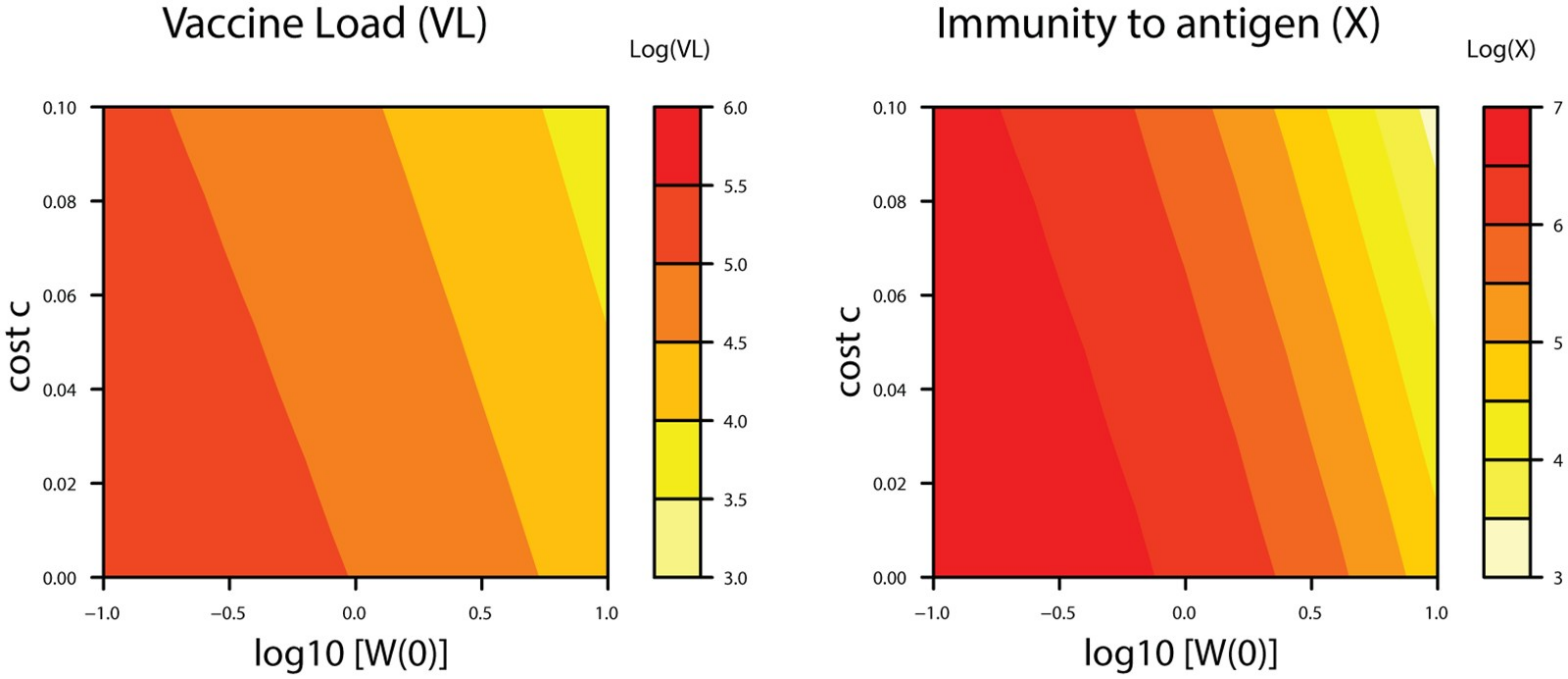
Viral load and the level of immunity to the vaccine antigen depend on evolution and vaccine composition (pre-host evolution). The final vaccine load and immunity against the vaccine antigen depends heavily on two parameters, the inoculum composition (plotted on the x-axis as initial abundance of the revertant virus, *W*(0)) and the growth advantage of the revertant within the host (*c*, plotted on the y-axis). The heat maps show how, as the composition shifts toward revertant or as vector superiority increases (as we move to the right or up), there is a reduction in the viral load of the vaccine (defined as *∫ V dt*, left panel) and in the magnitude of immunity to the vaccine antigen (*X*, right panel). The initial amount of vaccine virus is always V(0) = 1 (i.e. logV(0) = 0). Note that the graphs span high frequencies of revertant in the inoculum that should be easily avoided (log W(0) = 1, i.e. W(0) = 10 V(0))—if the researcher is alert to the possibility. We include such extremes merely to show that the outcome is relatively insensitive to small changes in vaccine composition. Equations, initial conditions and parameter values not shown here are given in S1 Appendix; R code is included in S1 File.

Illustrations of dynamics from individual trials convey many details. However, without a specific empirical basis for the parameter values chosen, the details have little assured relevance. We therefore provide contour plots that allow easy comparison of many different trials in which parameters of specific interest are varied (Fig 5). These graphs show the cumulative vaccine load (left panel) and final level of immunity to vaccine antigen (right) as a function of initial revertant frequencies and selective advantage of the revertant (*c*). A strong correspondence exists between vaccine load and the level of immunity generated, as is observed empirically following infection [40]. Subsequent figures therefore illustrate the level of immunity. The initial composition of the inoculum matters somewhat more to the adaptive response than does the intrinsic cost of the vaccine (as evident by the contours being closer to vertical rather than horizontal), but this pattern rests heavily on the parameter ranges chosen. Indeed, unrealistically large values of initial revertant levels (*W*(0)) are illustrated to offer contrast, as the outcomes are otherwise moderately insensitive to vaccine composition in these graphs. The good news is that, when the inoculum is mostly vaccine and revertant fitness is not high, evolution has little effect on viral load or final level of immunity (i.e., the lower left of each panel has a broad area of one color). This occurs because of the short duration of infection. Over longer periods of vaccine growth, the selective advantage of the revertant would undoubtedly play an increasing role in evolution. The large number of parameters (13) limits the degree to which we can conduct comprehensive sensitivity tests, so the trials are confined to variations in those parameters of greatest interest.

#### Vaccine evolution driven by adaptive immunity

We focus on infections of short duration—that are cleared and do not rebound once suppressed. Factors that limit the duration of infection include resource limitation, and innate and adaptive immunity. For the most part these factors act equally against vaccine and revertant virus. Only one factor, adaptive immunity to the vaccine antigen (*X*), acts specifically on the vaccine virus and not the revertant. Intuition suggests that this adaptive immunity to the antigen can potentially suppress the vaccine’s growth and give an advantage to the revertant. As with intrinsic fitness costs, this selection might feed back to limit vaccine growth and thus limit the development of further immunity to antigen by allowing revertant to grow and interfere with vaccine. This section considers whether these arguments are supported by the model.

Any real vaccine that elicits immunity against the antigen may also experience an intrinsic fitness cost. The effect of immunity on evolution would then be confounded with the effect of intrinsic fitness effects on evolution, making it difficult to isolate one from the other. The models do not face this problem, however. They can be parameterized so that the only possible selection against the vaccine comes from immunity (by setting *c* = 0). Vaccine populations can also be freed of revertant by omitting revertant from the inoculum and setting the mutation rate to 0. Thus, we can measure the effect of adaptive immunity on vaccine growth from trials that lack revertant and then compare those results with trials that include revertant.

There are several background points to note about the model structure. First, adaptive immunity specific to vaccine (*X*) develops at a rate proportional to the vaccine abundance (*V*) and parameters *s* (rate of clonal expansion of adaptive immunity) and *ϕ*_*X*_ (antigen concentration yielding half the maximum growth rate of adaptive immunity *X*). In contrast, the impairment specific to vaccine is due to the level of immunity (*X*) and the vaccine impairment parameter *k*_*X*_. Thus, immunity can develop while imposing little or no impairment, i.e., when *k*_*X*_ → 0. Second, adaptive immunity to the vector (*Y*) develops according to its own growth rate parameter (*ϕ*_*Y*_) in response to vaccine plus revertant abundance (*V* + *W*), and it impairs both vaccine and revertant growth equally by impairment parameter *k*_*Y*_. When revertant is present, it increases the level of immunity to vector backbone/revertant but does not directly affect immunity specific to the vaccine. This immunity will result in faster clearance of both revertant and vaccine, and this results in decreased immunity to the antigen; this is the ‘interference’ that causes a problem from vaccine evolution.

**Fig 6:**
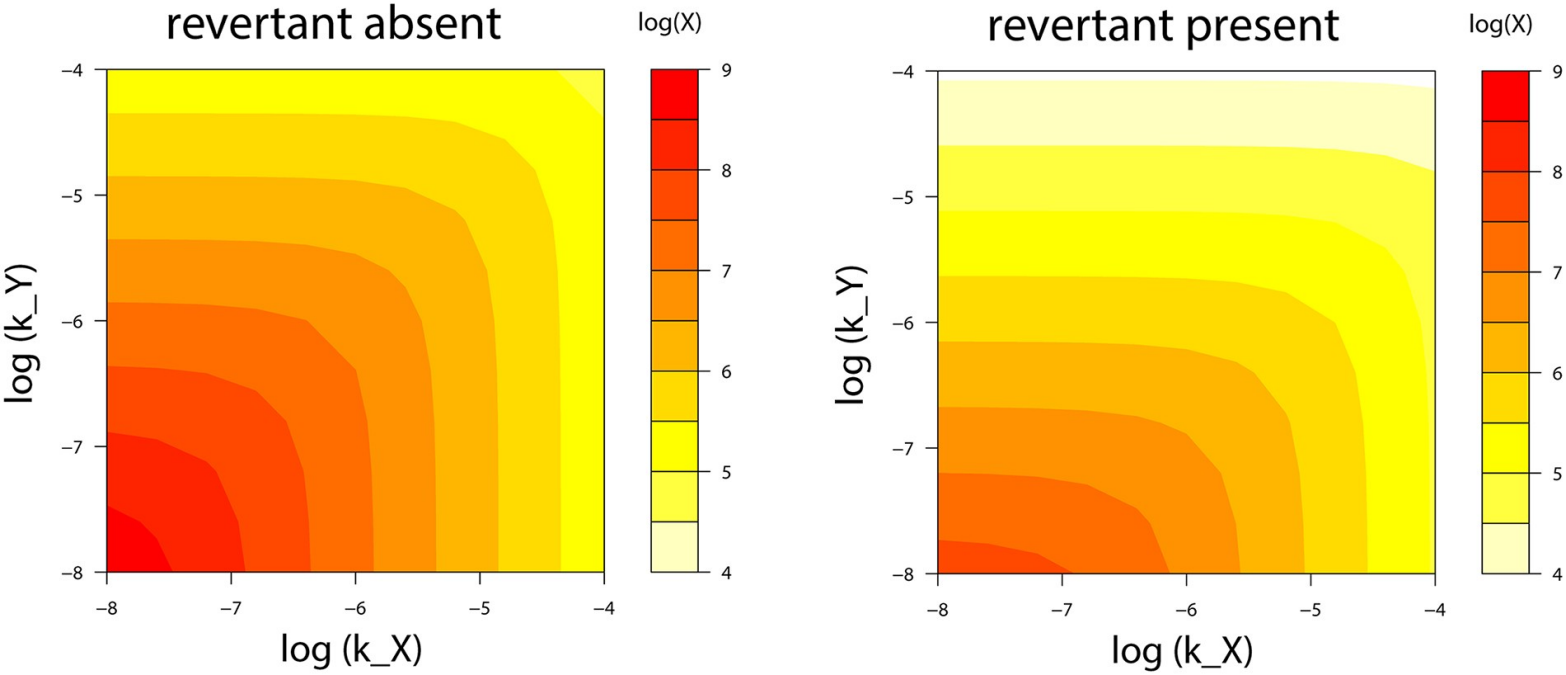
Effect of evolution on the suppression of immunity by impairment parameters. The final level of immunity to the vaccine antigen (*X*) depends heavily on the inhibitory parameters *k*_*X*_ and *k*_*Y*_—which respectively describe how strongly immunity to the vaccine (*X*) and revertant (*Y*) suppress the viral populations. The left plot considers the absence of revertant, hence no evolution. The right panel introduces revertant at 3/4 the inoculum, with the same total inoculum size as in the left panel. The revertant reduces immunity *X*, but the effect of increasing *k*_*X*_ is not made worse by the revertant. Intrinsic fitness differences are absent; mutation rate of vaccine to revertant is set to 0 and fitness benefit of revertant (*c*) is 0. Equations, initial conditions and parameter values not shown here are given in S1 Appendix; R code is included in S1 File.

Trials were run that contrasted revertant absence versus revertant introduced at 75% of the inoculum—no evolution versus primairly pre-host evolution, respectively (Fig 6). Absence of the revertant is the baseline against which the effect of evolution can be compared. The horizontal axis varies *k*_*X*_, the parameter for impairment/killing specific to vaccine, and the vertical axis varies *k*_*Y*_, impairment to vector, which affects vaccine and revertant equally. In both panels, increasing impairment against vaccine leads to lower levels of immunity to the vaccine—this is the self-limiting effect of adaptive immunity, which exists even in the absence of evolution. As expected, impairment of immunity to vaccine by immunity to vector is also found.

A large effect of inoculum composition on vaccine immunogenicity is evident by comparing the left panel (no mutation, no evolution) and right panel (chiefly prehost evolution): introduction of revertant reduces the level of immunity against vaccine up to 10-fold. For the right panel, the revertant is 3/4 the inoculum and has no intrinsic advantage over vaccine; total inoculum size is unchanged. All reduction of immunity against vaccine is thus due to revertant in the inoculum and any within-host evolution from the selective effect that stems from immunity against vaccine. Of the two parameters, *k*_*Y*_ has a much larger effect than does *k*_*X*_: compared to the left panel, the right panel, with revertant present, has vaccine-specific immunity suppressed more than an order of magnitude along the *k*_*Y*_ axis, much less so on the *k*_*X*_ axis. We attribute this effect of *k*_*Y*_ to interference by the revertant: the revertant elicits high levels of immunity (*Y*) that indiscriminately also suppress vaccine, thereby suppressing vaccine-specific immunity *X*. The magnitude of interference depends not only on revertant abundance but also on the values of *k*_*X*_, *k*_*Y*_, and the innate immune response (*k*_*Z*_), so interference can appear more or less important in other trials using the same revertant abundance.

A question motivating this analysis was one step deeper in the complexity of these effects: does the self-limiting effect of adaptive immunity worsen when revertant is present? This question can be answered by comparing the self-inhibitory effect between left and right panels as *k*_*X*_ is increased. By inspection of colors along the horizontal axes, it is seen that the self-inhibitory effect is actually somewhat reduced by the revertant. The revertant lowers the overall response to vaccine, but when correcting for that difference, the effect of increasing *k*_*X*_ is slightly weaker in the right panel than in the left. We attribute this weakening of self-limitation as due to vaccine levels being increasingly controlled by immunity against revertant.

In sum, therefore, immunity to the vaccine (*X*) is reduced by itself (depending on the immunity parameter, *k*_*X*_) and by revertant. The two effects do not interact to make the problem worse than from their separate effects.

### Escaping the effects of evolution: Manipulate the inoculum

**Fig 7:**
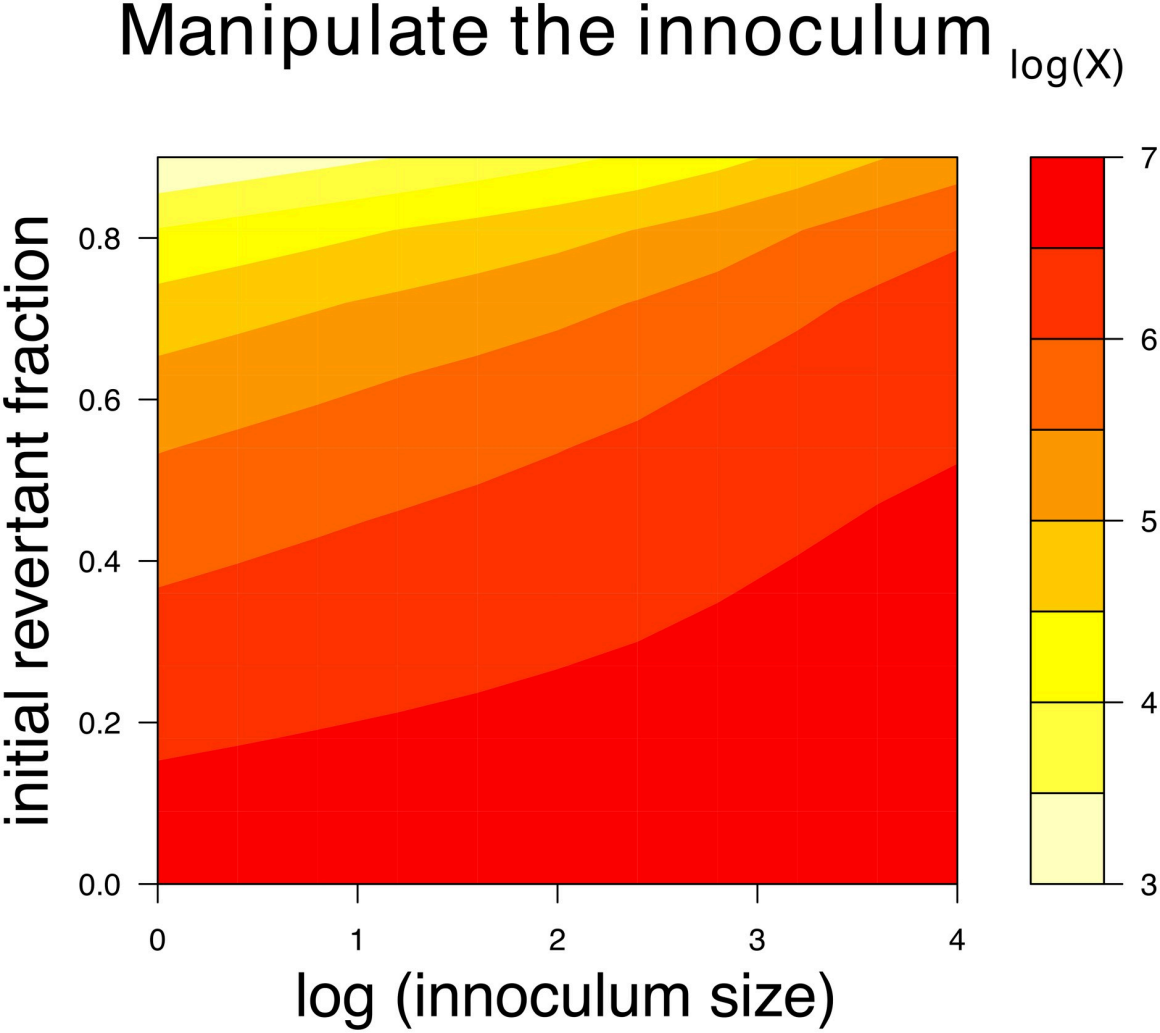
Effects of manipulating the inoculum on immunity to the vaccine. Small inocula that contain vaccine plus revertant are more prone to reduced immunity levels than are large inocula with little revertant. Composition of the vaccine has the larger effect for the inoculm sizes and initial revertant fractions shown, as indicated by the contours being more horizontal than vertical. An intrinsic fitness cost of *c* = 0.1 was set for these trials. Smaller *c* values would lead to higher vaccine and immunity levels across the graphs. Equations, initial conditions and parameter values not shown here are given in S1 Appendix; R code is included in S1 File.

The results above suggest that vaccine evolution is only likely to compromise immunity if there is substantial pre-host or within-host evolution and if this evolution depresses vaccine virus in the host. As the short duration of infection limits within-host evolution, one means of achieving vaccine efficacy is to control the inoculum. Two ways of controlling the inoculum are to control its composition and to control its size. Pre-host evolution can be reversed by purifying the inoculum after the fact or by taking care to start with a pure isolate and limiting growth (e.g., Fig 1). The benefit of suppressing revertant frequency in the inoculum is evident in Fig 7: the magnitude of immunity to the vaccine increases by orders of magnitude as the initial frequency of the revertant is decreased. The effect is strongest at low inoculum levels, pointing to the other solution—increase inoculum size.

Intuition also suggests that the deleterious effects of evolution can be reduced by increasing the inoculum size, provided the composition does not change: to achieve a threshold antigen level, a large inoculum requires less growth than a small one. Less growth reduces the potential for evolution—in the extreme, a large enough inoculum requires no vaccine growth, as with killed vaccines. These conjectures are supported by Fig 7: when the revertant frequency in the inoculum is high, increasing the inoculum size appreciably increases the magnitude of immunity; a much reduced benefit is seen when revertant frequency is low, likely because there is less evolutionary interference from the revertant. These results suggest parallel benefits from reducing the frequency of the revertant in the inoculum and increasing the dose. Consideration of the gains from each could help choose an economically feasible strategy, since both purifying the inoculum and increasing its dose are likely to incur financial costs.

Whether and how well controlling the inoculum will work in practice will depend on details. Solutions may be quantitative rather than absolute. Intuition is useful for guidance but needs to be confirmed by formal analyses, guided by data from the specific implementation.

## Discussion

Any live viral vaccine may evolve within the host. The potential for attenuated viruses to revert to wild-type virulence is well appreciated [1, 2], even if it presents a problem for relatively few vaccines (e.g., attenuated polio, [41]). There is also a potential for live, recombinant vector vaccines to evolve—our focus in this paper—with the main concern being loss or reduced expression of the transgenic insert [4, 42]. If such a vaccine were to evolve fast enough or long enough that it lost the insert, vaccine efficacy might well suffer. We find that evolution during manufacture (pre-host evolution) can play a more important role than within-host evolution in reducing vaccine efficacy, and furthermore that it may be the more easily mitigated.

We developed and analyzed models to explore ways in which vaccine evolution could lead to a reduction in vaccine efficacy. An intrinsic fitness advantage of the revertant virus, expected because transgene expression is likely to have metabolic and other costs, will lead to vaccine being gradually overgrown by revertant. This is only likely to cause a reduction in the immunity to the vaccine antigen if it leads to a reduction in the absolute amount (as opposed to merely a reduction in relative frequency) of the vaccine virus. There are in fact several mechanisms by which an ascending revertant population may suppress vaccine: revertant can reduce the amount of the vaccine virus in the host if the revertant uses resources required for virus replication or if the vaccine virus is cleared by the innate or adaptive responses elicited by the revertant.

The clear and positive message from our study is that vaccine evolution, if it proves to be a problem for immunization, should be easily mitigated by manipulating the vaccine inoculum. Critical to understanding and addressing this problem is recognizing that the vaccine may evolve both within the host and also during manufacture, whereby the inoculum already carries modest to high levels of revertant. The composition of the inoculum can have a large effect on within-host evolution and immunity. By limiting the amount of revertant in the inoculum, and also by boosting the inoculum level, it should usually be possible to limit the amount of within-host vaccine evolution and ensure that immunization is effective. We emphasize, however, that this solution will typically not work for transmissible vaccines and vaccines that establish long term infections within the host. Furthermore, using a large inoculum may seem to defeat the purpose of using a live vaccine.

There may be cases in which vaccine evolution is so rapid that controlling the inoculum is not sufficient. The solution in this case is to develop or engineer the vaccine with less of a disadvantage. The timing and tissues of antigen expression, location of the transgene in the vector genome, and the size of the transgene may all influence intrinsic fitness effects [10, 11, 19, 43, 44]. Directed evolution approaches might also improve vaccine efficacy: one simple approach in reducing an intrinsic cost might be to adapt the vector *in vitro* to host cells expressing the antigen *in trans*, allowing compensatory mutations to evolve in response to the antigen before the transgene is cloned into the genome. This adapted vector would then be used as the vaccine backbone. Another simple approach would be to compete several different vaccine designs *in vitro* and pick the design with highest retention of the transgene. Any approach using *in vitro* adaptation needs to avoid adapting the vector to the extent that it compromises ability to grow *in vivo*. Most of these possibilities are ways to reduce pre-host evolution and reduce revertant concentration in the inoculum. One may hope that vaccine designs which reduce pre-host evolution also reduce within-host evolution.

Measuring the intrinsic fitness effect of the transgene is likely to be an important step in vaccine design. For assessing vaccine evolution, the relevant biological realms are within the host and *in vitro*. *In vitro* growth environments are the more easily studied and may reveal much about a vaccine’s intrinsic propensity to evolve loss of antigen expression. There are various ways intrinsic fitness effects and their evolutionary consequences might be studied. Vaccine growth in tissue culture may reveal some aspects of intrinsic fitness effects and should be relatively easy to study. Deletion of the transgene *per se* would be detectable by PCR, and the fitness advantage of revertant over vaccine could be measured from changes in revertant frequency. The quantitative relevance of an *in vitro* estimate to *in vivo* growth would be unknown, but the measure should allow qualitatively comparing engineering designs that improve intrinsic vaccine fitness. If vaccine reversion were due to down regulation of the transgene instead of deletion, fitness estimation would require knowing the mutations responsible and monitoring their frequencies. Use of culture-wide antigen levels to measure fitness might provide a sense of whether vaccine evolution would lead to reduced antigen levels *in vivo*, but it would be less sensitive in measuring evolution than is measuring mutation frequencies.

Evolution is not the only consideration in designing a recombinant vector vaccine, and the model helps us identify vaccine properties that promote efficacy. First the vaccine should elicit an immune response that rapidly clears the pathogen (i.e. the rate constant for clearance of the pathogen, call it *k*_*P*_, is high). Second, the vaccine should elicit a large response to this antigen. This requires that the antigen rapidly elicits immunity (i.e. has low *ϕ*_*X*_, and in terms of immunology it should be an immunogenic antigen), and also requires a high vaccine viral load to generate a large response. Engineering this requires tackling a trade-off between avoiding vaccine clearance (i.e. having a low *k*_*X*_) but allowing for rapid clearance of the pathogen (having a high *k*_*P*_). Vaccines designed to express the antigen in a form that is different from that in the pathogen might help solve this problem. Thus, to elicit immunity to influenza, one might design secreted forms of the hemagglutinin or neuraminidase proteins. A recombinant hemagglutinin protein that is secreted rather than on the virion surface would prevent the antibody response to this protein from clearing the recombinant vector vaccine (have low *k*_*X*_) without compromising the clearance of the influenza virus pathogen which has hemagglutinin on its surface (i.e. has high *k*_*P*_). In this manner our model allows the identification and tuning of parameters that affect vaccine efficacy, and a comprehensive search of parameter space would identify ideal combinations of vaccine properties.

*In vitro* assays may be useful in measuring intrinsic fitness effects, but *in vivo*—in the patient—is the ultimate environment for studying within-host evolution and its effects. Not only are the dynamics of viral spread different between *in vitro* and *in vivo* environments, but most immune components will be in play only *in vivo*. Furthermore, those components may vary across tissues within the host. Sampling across this heterogeneity *in vivo* will be challenging but may be necessary to know whether, when, and where vaccine evolution is a problem. If revertant remains a minority of the population, we expect that vaccine evolution can be ignored. Perhaps *in vitro* studies of vaccine evolution will provide most of the information relevant to *in vivo* evolution, but it is too early to know.

We have focused on recombinant vector vaccines that cause acute infections. Necessarily, our recommendations are based on simple models that are caricatures of the complex within-host dynamics of acute infections. Simple models are appropriate at this stage because of uncertainties at many biological levels, and under these circumstances simple models frequently generate more robust results than do complex models [45, 46].

The generation of innate and adaptive responses can be modeled with different assumptions than used here, and those alternative processes may affect the conclusions. For example, time-lags in the activation of cells may dominate the time for the generation of an innate immune response, with virus density having a consequently smaller role than assumed here (as can be seen in [47] and modeled in [30]). We have modeled that responses to different antigens are generated independently of each other and do not compete. We have done so because vaccines are likely to cause relatively mild infections during which the densities of pathogen and immune cells do not reach sufficiently high levels required for competitive interactions to be important. The adaptive immune response may be more influenced by recruitment which is followed by a period of proliferation even in the absence of antigen [48–50]. Both these scenarios would minimize the impact of evolutionary changes in the vaccine on the amount of immunity generated to the transgene.

Finally, it is easily appreciated that there are realms we do not consider, such as within-host spatial structure [51] and recombinant vector vaccines based on viruses such as cytomegalovirus that cause persistent infections [52] or that are transmissible. Spatial structure may limit the impact of vaccine evolution on immunity (e.g., prevent mutants from taking over the entire population). In contrast, vaccines that cause persistent infections or are transmissible are likely to be more severely affected by evolution than are vaccines causing acute infections, as there is a longer timeframe for evolution to operate.

With so little experience from recombinant vector vaccines, we can merely guess how commonly the neglect of within-host evolution will compromise vaccine efficacy. Given that simple steps can be taken to reduce vaccine evolution, vaccine development programs should at least entertain the possibility that evolution can underlie failure. Avoiding vaccine evolution may be easier than developing an entirely new vaccine.

## Supporting information

S1 Appendix Equations and parameters. (PDF)

S1 File R Markdown file of code to run numerical trials of equations and generate figures. This file was used to generate the figures in the paper but may also be used to run other conditions, such as the case when resource limitation controls the infection.
(RMD)

S2 File Mathematica code to run numerical trials of equations. (NB)

S1 Fig Additional figures generated by the existing S1 File. The S1 File allows easy modification to explore other parameter values, so the figures generated here do not represent a thorough coverage of parameter space.
(PDF)
