# Supplementary material for: Recombinant vector vaccine evolution
Source: PLoS Comput Biol. 2019 Jul 19;15(7):e1006857. doi: 10.1371/journal.pcbi.1006857 (PMC6668849; doi:10.1371/journal.pcbi.1006857)
Supplement: S1 Appendix — (PDF) [file pcbi.1006857.s001.pdf]

S1 Appendix to: Recombinant vector vaccine evolution

James J. Bull, Scott L. Nuismer and Rustom Antia

The models: equations and parameters

The models used here specify features of viral infections. Some basics include the following:

1. Two viral types: Only vaccine and wild-type (vector or revertant) are ever present.

2. Acute infections. Infections are short term because they are subject to control and clearance by any combination of three factors: resource limitation, innate immunity and adaptive immunity.

Further details not included in this Appendix can be found in Supplements.

The following table defines the variables and parameters used in these equations.

**Term**	**Description**	**Initial Value**
V	Vaccine	1 (rescaled)
W	Wild-type (revertant) virus	1 (rescaled)
R	Resource	∞ for innate control
X	Adaptive response to vaccine antigen	1 (rescaled)
Y	Adaptive response to revertant backbone	1 (rescaled)
Z	Innate immune response (percent max.)	0

Table 1: Variables and initial conditions

Equations

Resources start with a fixed amount and are depleted by vaccine and revertant growth, without replenishment. We rescale resource so one unit of resource gets converted to one unit of vaccine virus and (1+c) units of the revertant:

**Term**	**Description**	**Value**
r	rate of growth of V	3 day^{-1}
c	cost to having recombinant antigen	$0 < c < 1$
d	death rate of virus	1 day^{-1}
μ	mutation rate for V to W	0 virus^{-1} day^{-1}
σ	rate of stimulation of innate immunity	2.7×10^{-5} virus^{-1} day^{-1}
k_Z	killing rate of V and W due to Z (innate immunity)	3×10^{-2} Z^{-1} day^{-1}
d_Z	decay of innate immunity in absence of antigen	1 day^{-1}
s	rate of clonal expansion of adaptive immunity	3 day^{-1}
ϕ_X	antigen abundance for half max growth of adaptive immunity X	10^3 virus
ϕ_Y	antigen abundance for half max growth of adaptive immunity Y	10^3 virus
k_X	killing rate of V due to X (immunity to insert)	10^{-6} X^{-1} day^{-1}
k_Y	killing rate of V and W due to Y (immunity to vector)	10^{-6} Y^{-1} day^{-1}

Table 2: Parameters

$$\text{(resource)} \quad \frac{dR}{dt} = \underbrace{-rV\frac{R}{\phi_R + R}}_{\text{resource for virus growth}} - \underbrace{r(1+c)W\frac{R}{\phi_R + R}}_{\text{resource for revertant growth}} .$$

The vaccine virus grows on resource R at rate r, depleted by mutation, death, and all 3 types of immunity:

$$\text{(vaccine virus)} \quad \frac{dV}{dt} = \underbrace{rV\frac{R}{\phi_R + R}}_{\text{virus growth}} - \underbrace{\mu V}_{\text{mutation to revertant}} - \underbrace{dV}_{\text{virus decay}} - \underbrace{(k_X X + k_Y Y + k_Z Z)V}_{\text{virus loss due to X, Y and Z}} .$$

Revertant grows on resource R at rate r(1+c), depleted by mutation, death, and 2 types of immunity (not X):

$$\text{(revertant virus)} \quad \frac{dW}{dt} = \underbrace{r(1+c)W\frac{R}{\phi_R + R}}_{\text{revertant growth}} + \underbrace{\mu V}_{\text{mutation}} - \underbrace{dW}_{\text{revertant decay}} - \underbrace{(k_Y Y + k_Z Z)W}_{\text{revertant loss due to Y and Z}} .$$

Adaptive immunity specific to vaccine grows according to its present value and a discounted value of the current vaccine density:

$$\text{(vaccine immunity)} \quad \frac{dX}{dt} = \underbrace{sX\frac{V}{\phi_X + V}}_{\text{clonal expansion of X}} .$$

Adaptive immunity common to vaccine and revertant grows according to its present value and a discounted value of the current vaccine plus revertant densities:

$$\text{(revertant immunity)} \quad \frac{dY}{dt} = \underbrace{sY\frac{V+W}{\phi_Y+V+W}}_{\text{clonal expansion of Y}} \quad .$$

Innate immunity, also common to vaccine and revertant, grows according to current levels of vaccine and revertant, with diminishing growth as a limit is approached. Innate immunity also decays:

$$\text{(innate immunity)} \quad \frac{dZ}{dt} = \underbrace{\sigma(V+W)(100-Z)}_{\text{activation of inactive cells}} - \underbrace{d_Z Z}_{\text{inactivation of Z}} \quad .$$

These models follow the usual assumptions of SIR models, except that susceptible hosts (host cells in our case) are modeled as Resource. As is typical in these models, the free virus and infected cells are assumed to be proportional, an assumption based on the rapid turnover of free virus (the quasi-steady state approximation, as per ref. 25 in the main document).
