# Supplementary material for: Recombinant vector vaccine evolution
Source: PLoS Comput Biol. 2019 Jul 19;15(7):e1006857. doi: 10.1371/journal.pcbi.1006857 (PMC6668849; doi:10.1371/journal.pcbi.1006857)
Supplement: S1 Fig — The S1 File allows easy modification to explore other parameter values, so the figures generated here do not represent a thorough coverage of parameter space. (PDF) [file pcbi.1006857.s004.pdf]

Supplementary figure to: Recombinant Vector Vaccine Evolution

James Bull and Scott L. Nuismer and Rustom Antia

04/VI/2019

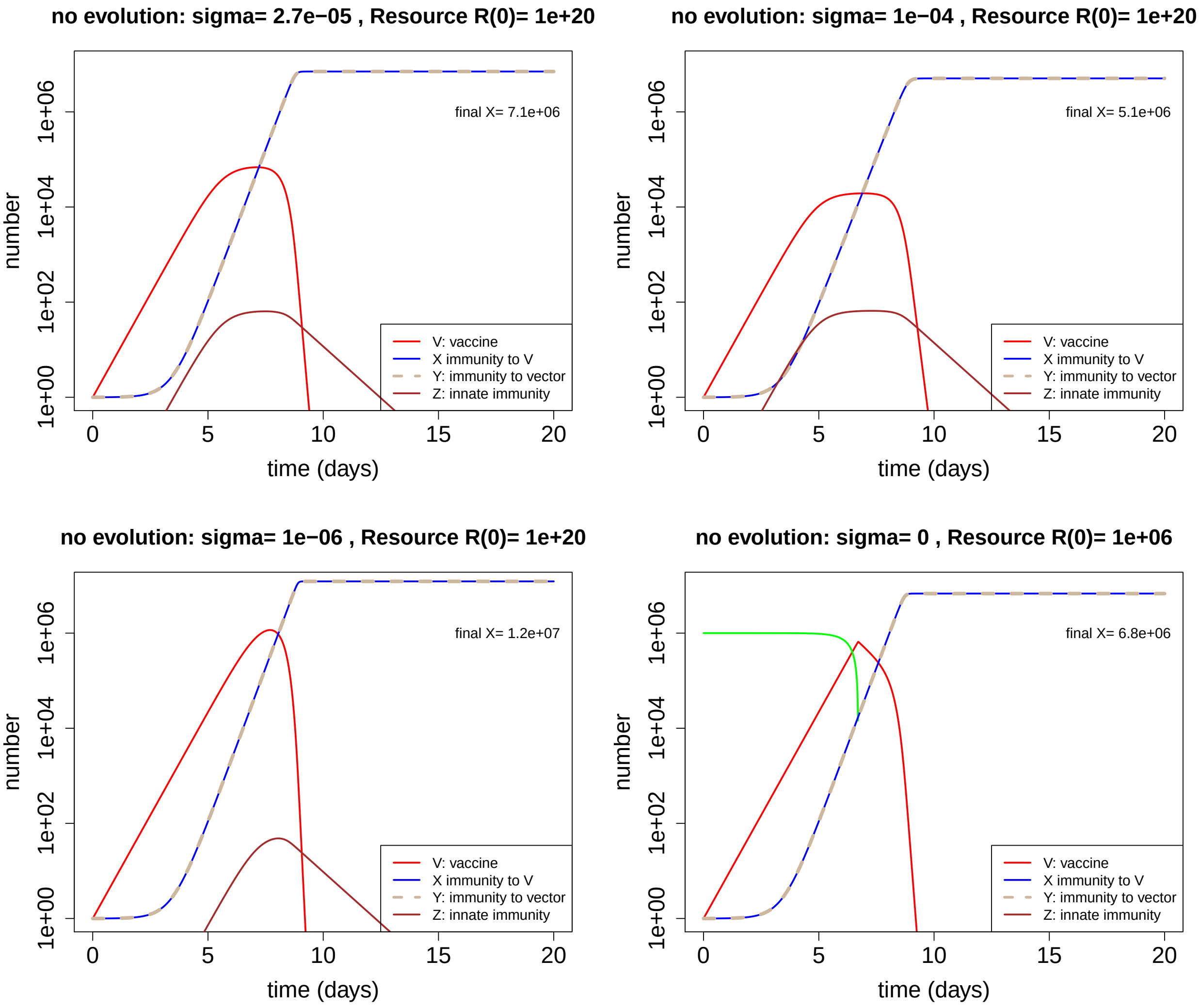

Figure 1: Dynamics with different values of σ and for Resource limitation (lower right panel)
